# Supplementary material for: GWAS meta-analysis of intrahepatic cholestasis of pregnancy implicates multiple hepatic genes and regulatory elements
Source: Nat Commun. 2022 Aug 17;13:4840. doi: 10.1038/s41467-022-29931-z (PMC9385867; doi:10.1038/s41467-022-29931-z)
Supplement: Supplementary file 1 — Supplementary Information [file 41467_2022_29931_MOESM1_ESM.pdf]

Supplementary information

GWAS Meta-Analysis of Intrahepatic Cholestasis of Pregnancy Implicates Multiple Hepatic Genes and Regulatory Elements

Peter H. Dixon[1*], Adam P. Levine[2,3*], Inês Cebola[4*], Melanie M. Y. Chan[2], Aliya S. Amin[1], Anshul Aich[2], Monika Mozere[2], Hannah Maude[4], Alice L. Mitchell[1], Jun Zhang[2,5], NIHR BioResource[6], Genomics England Research Consortium[7], Jenny Chambers[8,9], Argyro Syngelaki[10], Jennifer Donnelly[11], Sharon Cooley[11], Michael Geary[11], Kypros Nicolaides[10], Malin Thorsell[12], William M. Hague[13], Maria Cecilia Estiu[14], Hanns-Ulrich Marschall[15], Daniel P. Gale[2*], Catherine Williamson[1*].

Affiliations
1. Department of Women and Children's Health, School of Life Course Sciences, King's College London, London, UK.
2. Department of Renal Medicine, University College London, London, UK.
3. Research Department of Pathology, University College London, London, UK.
4. Section of Genetics and Genomics, Department of Metabolism, Digestion and Reproduction, Imperial College London, London, UK.
5. Division of Nephrology, Department of Medicine, Third Affiliated Hospital of Sun Yat-Sen University, Guangzhou, Guangdong, China.
6. NIHR BioResource, Cambridge University Hospitals, Cambridge Biomedical Campus, Cambridge, UK.
7. Genomics England, Queen Mary University of London, UK.
8. ICP Support, 69 Mere Green Road, Sutton Coldfield, UK.
9. Women's Health Research Centre, Imperial College London, London, UK.
10. Harris Birthright Research Centre for Fetal Medicine, King's College Hospital, London, UK.
11. The Rotunda Hospital, Dublin, Ireland.
12. BB Stockholm, Danderyd Hospital, Stockholm, Sweden.
13. Robinson Research Institute, The University of Adelaide, South Australia, Australia.
14. Ramón Sardá Mother's and Children's Hospital, Buenos Aires, Argentina.
15. Department of Molecular and Clinical Medicine/Wallenberg Laboratory, University of Gothenburg, Gothenburg, Sweden.

*Indicates equal author contribution

Correspondence: Catherine Williamson, 2.30W Hodgkin Building, Department of Women and Children's Health, School of Life Course Sciences, FOLSM, King's College London, Guy's Campus, London SE1 1UL, UK. Email: catherine.williamson@kcl.ac.uk

Table of contents

TABLE OF CONTENTS 2
SUPPLEMENTARY FIGURES 3
SUPPLEMENTARY FIGURE 1 3
SUPPLEMENTARY FIGURE 2 4
SUPPLEMENTARY FIGURE 3 5
SUPPLEMENTARY FIGURE 4 6
SUPPLEMENTARY FIGURE 5 7
SUPPLEMENTARY FIGURE 6 8
SUPPLEMENTARY FIGURE 7 9
SUPPLEMENTARY FIGURE 8 10
SUPPLEMENTARY FIGURE 9 11
SUPPLEMENTARY NOTE ON METHODS 12
NIHR BIORESOURCE RARE DISEASE COLLABORATION (NIHR-RD) 12
Study and cohort descriptions *12*
Whole-genome sequencing, variant calling and quality control *13*
Ancestry and relatedness estimation *13*
THE 100,000 GENOMES PROJECT (100KGP) 14
Study and cohort descriptions *14*
Whole-genome sequencing, variant calling and quality control *14*
Ancestry and relatedness estimation *15*
SUPPLEMENTARY INFORMATION REFERENCES 16

Supplementary Figures

Supplementary Figure 1

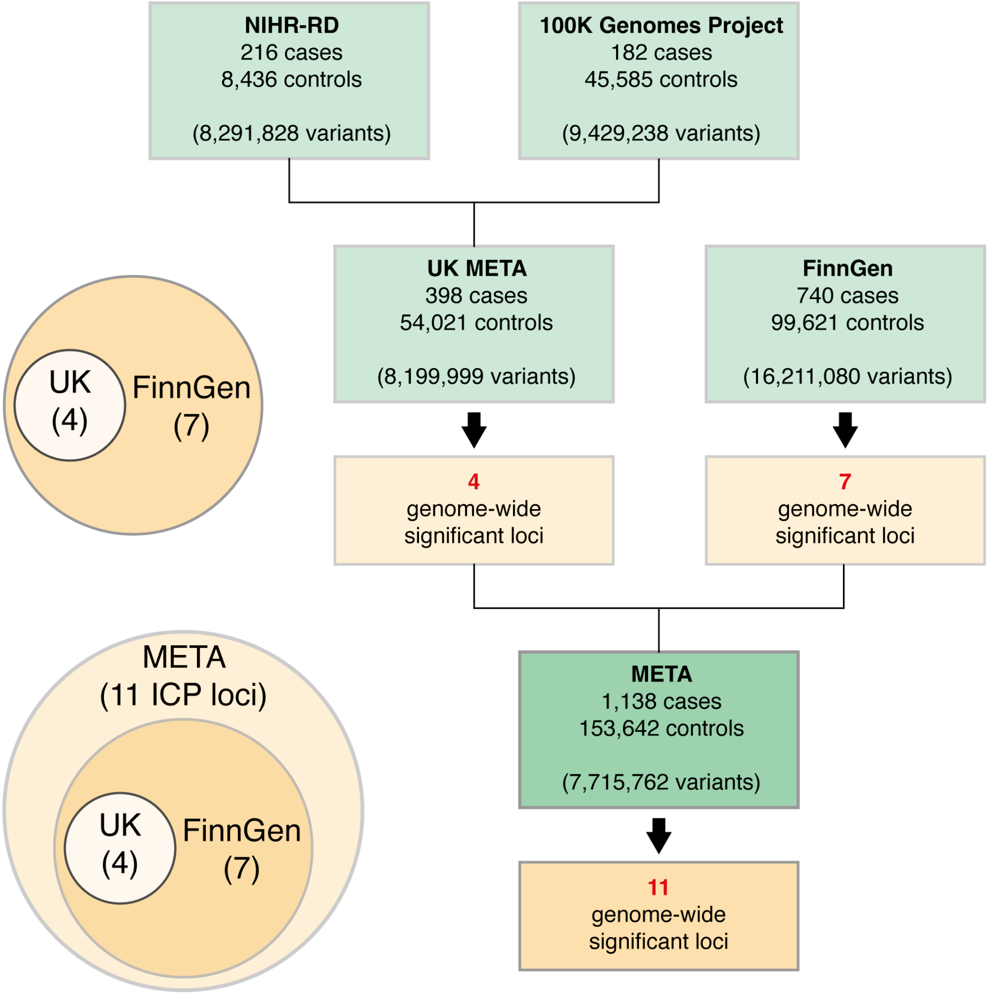

Study design and variant prioritization strategy. Genome-wide association study meta-analysis design. The number of cases, controls and variants examined and the number of genome-wide significant ($p < 5\times10^{-8}$) loci identified in each cohort are indicated. NIHR-RD refers to the NIHR BioResource Rare Disease collaboration cohort. The Venn diagrams on the left illustrate the number of genome-wide significant loci that were identified in the different cohorts/meta-analyses. 'UK' refers to the meta-analysis of the UK cohorts (NIHR-RD and 100K Genomes Project). 'META' refers to the combined meta-analysis of the UK meta-analysis with FinnGen.

Supplementary Figure 2

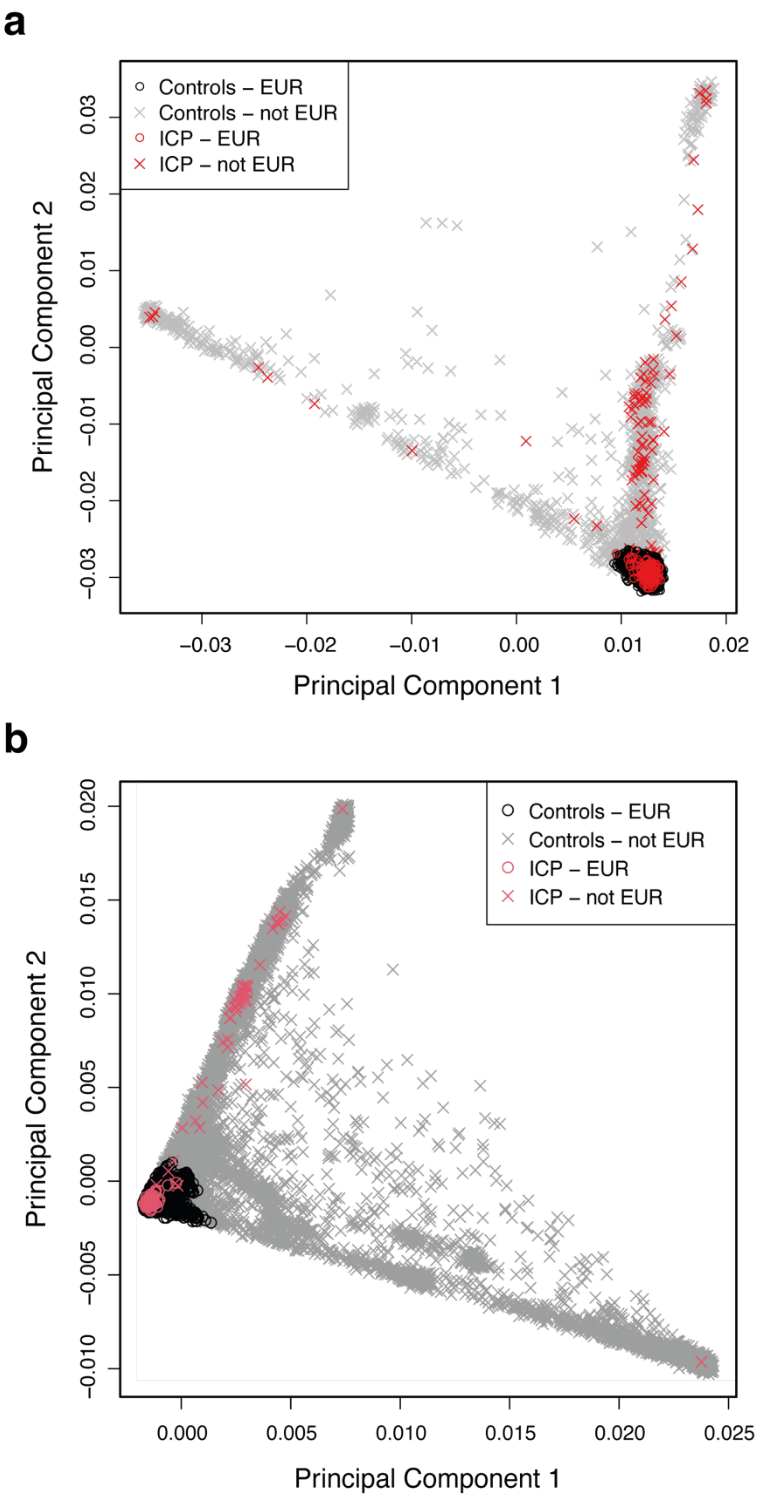

Principal component analysis. The first two principal components are shown for the NIHR BioResource Rare Disease collaboration cohort (NIHR-RD) (a) and the 100,000 Genomes Project cohort (b) highlighting intrahepatic cholestasis of pregnancy (ICP) cases (red) by ethnicity (European (EUR) circle, not European cross). European controls are indicated by black circles and not European controls are indicated by grey crosses. Only individuals of European ancestry (circles) were included in subsequent analyses.

Supplementary Figure 3

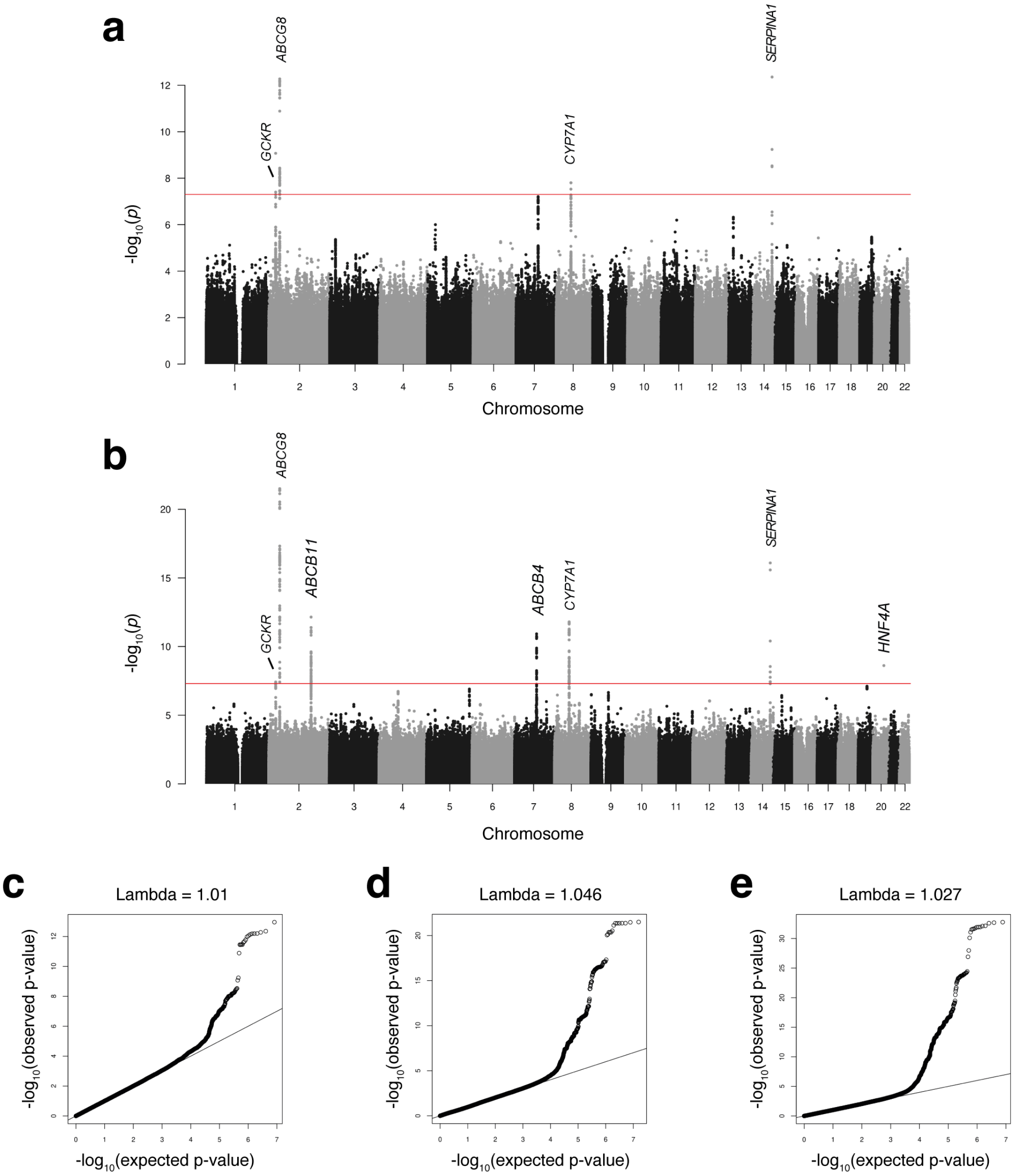

Genome-wide association study of intrahepatic cholestasis of pregnancy (ICP), UK meta-analysis and FinnGenn data. a, Manhattan plot for the genome-wide association study (GWAS) meta-analysis of ICP in the NIHR BioResource Rare Disease collaboration cohort (NIHR-RD) and 100,000 Genomes Project (100KGP) cohorts. All individuals are of European ancestry. Association testing was performed using a generalized logistic mixed model to account for population stratification and saddle-point approximation to control for type 1 error rates due to unbalanced case-control ratios, followed by meta-analysis weighting the effect size estimates using the inverse of the standard errors. The chromosomes are ordered on the x-axis; the y-axis shows the $-\log_{10}(P)$ values for the association tests. Four loci achieved genome-wide significance ($p < 5\times10^{-8}$, indicated by the red line). The prioritized gene at each locus that achieved significance in the replication meta-analysis cohort is shown. **b,** Manhattan plot for the GWAS of ICP in FinnGen (https://r4.finngen.fi/pheno/O15_ICP). Association testing was performed using a generalized logistic mixed model to account for population stratification and saddle-point approximation to control for type 1 error rates due to unbalanced case-control ratios. Seven loci achieved genome-wide significance. **c-e,** QQ plots showing the relationship between observed and expected $-\log_{10}(P)$ values from the GWAS meta-analysis of NIHR-RD and 100KGP (c), FinnGenn (d) and the combined meta-analysis (e). The genomic inflation (lambda) is shown in each graph.

Supplementary Figure 4

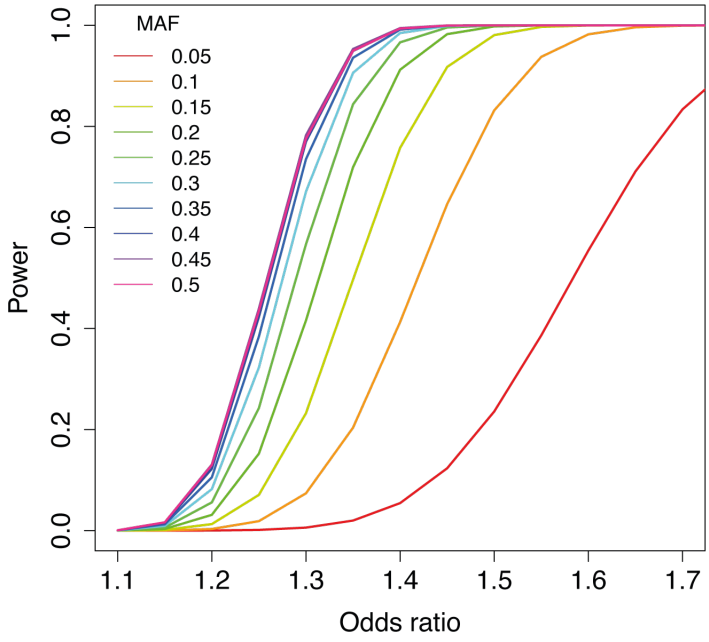

Post-hoc power calculations based on the size of the cohort utilised for the combined meta-analysis (1,138 cases and 153,642 controls). The power (y-axis) to detect association at $p<5\times10^{-8}$ is shown for varying odds ratio (x-axis) and minor allele frequencies (MAF) (coloured lines). Power calculations were performed using a logistic model under genetic additivity.

Supplementary Figure 5

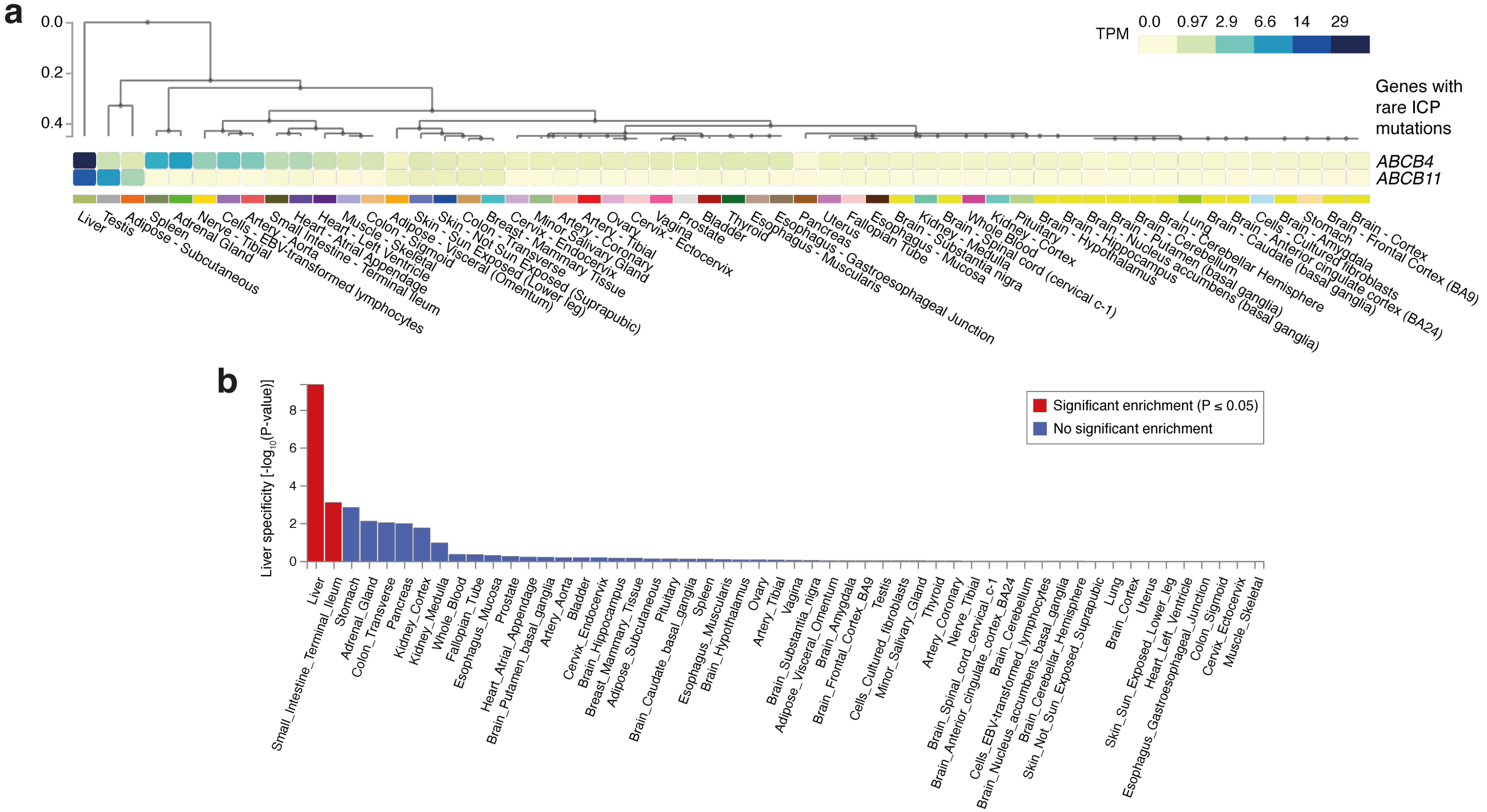

Liver-specificity of ICP genes. a, Clustering analysis of GTEx samples based on the mean expression levels of *ABCB4* and *ABCB11*, the two genes that most frequently harbour rare ICP causal mutations[1]. The clustering shows that the two genes are highly enriched in liver tissue in comparison with all available tissues. **b,** Tissue-based differential expression analysis from FUMA GENE2FUNC across GTEx tissues reveals that ICP susceptibility genes are enriched in liver tissue (identified as upregulated genes in liver versus other tissues). For this analysis, we considered a set of 35 genes, which corresponded to the nearest genes of all genome-wide significant ICP variants ($P < 5 \times 10^{-8}$) plus all variants in high LD (EUR $r^2 > 0.8$) (see Methods). Enrichment analysis was performed using hypergeometric testing with adjustment for multiple comparisons with Bonferroni correction. Significant enrichment at Bonferroni corrected $P \leq 0.05$ are coloured in red.

Supplementary Figure 6

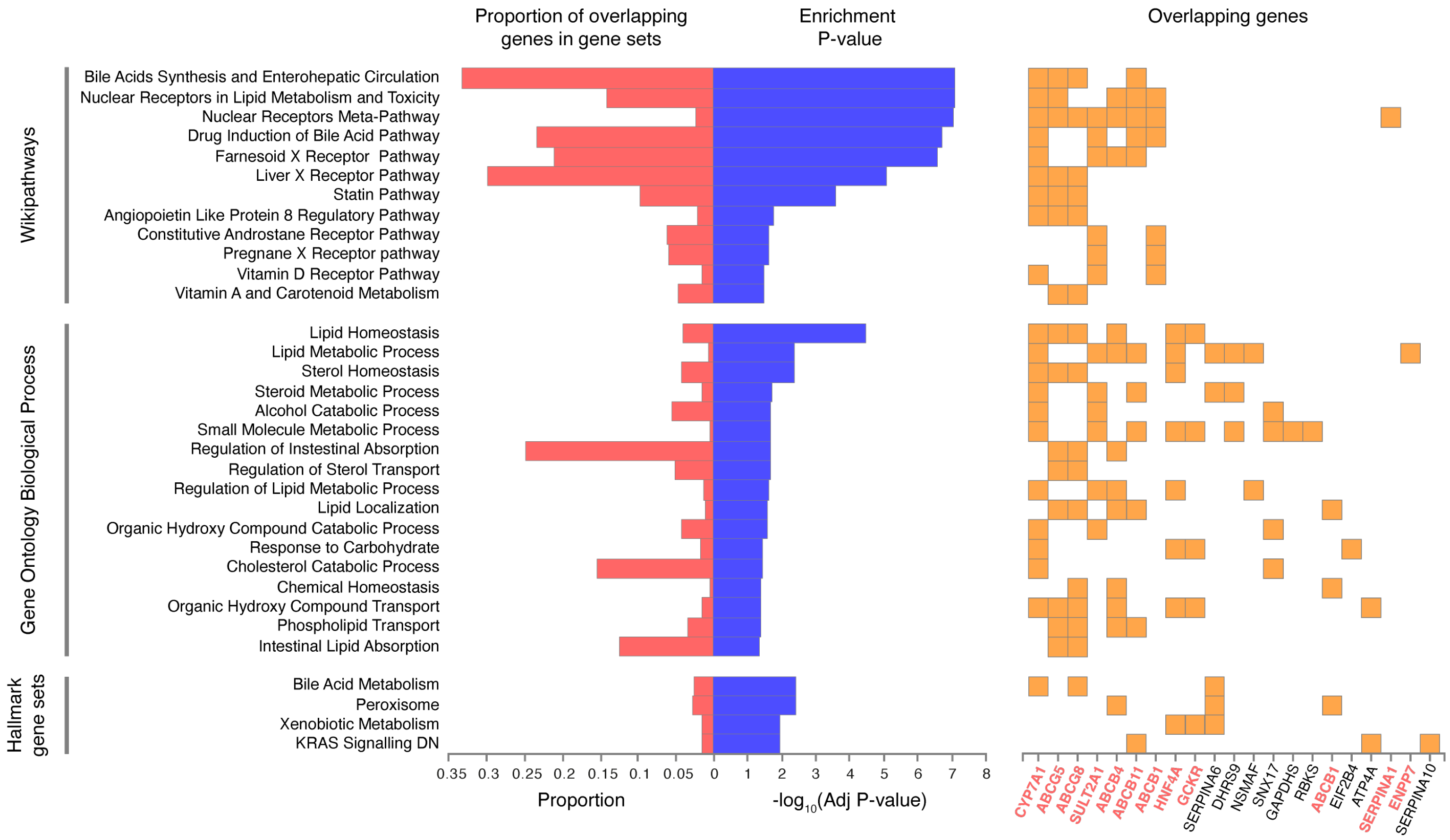

Pathway enrichment analysis of ICP susceptibility genes. The analysis was undertaken using FUMA GENE2FUNC. Wikipathways, Gene ontology biological process and Hallmark gene sets were examined. Genes were selected for inclusion as per Supplementary Figure 5. The genes highlighted in red were prioritised as likely causal of ICP susceptibility after functional genomic analyses. Enrichment analysis was performed using hypergeometric testing with adjustment for multiple comparisons with Bonferroni correction.

Supplementary Figure 7

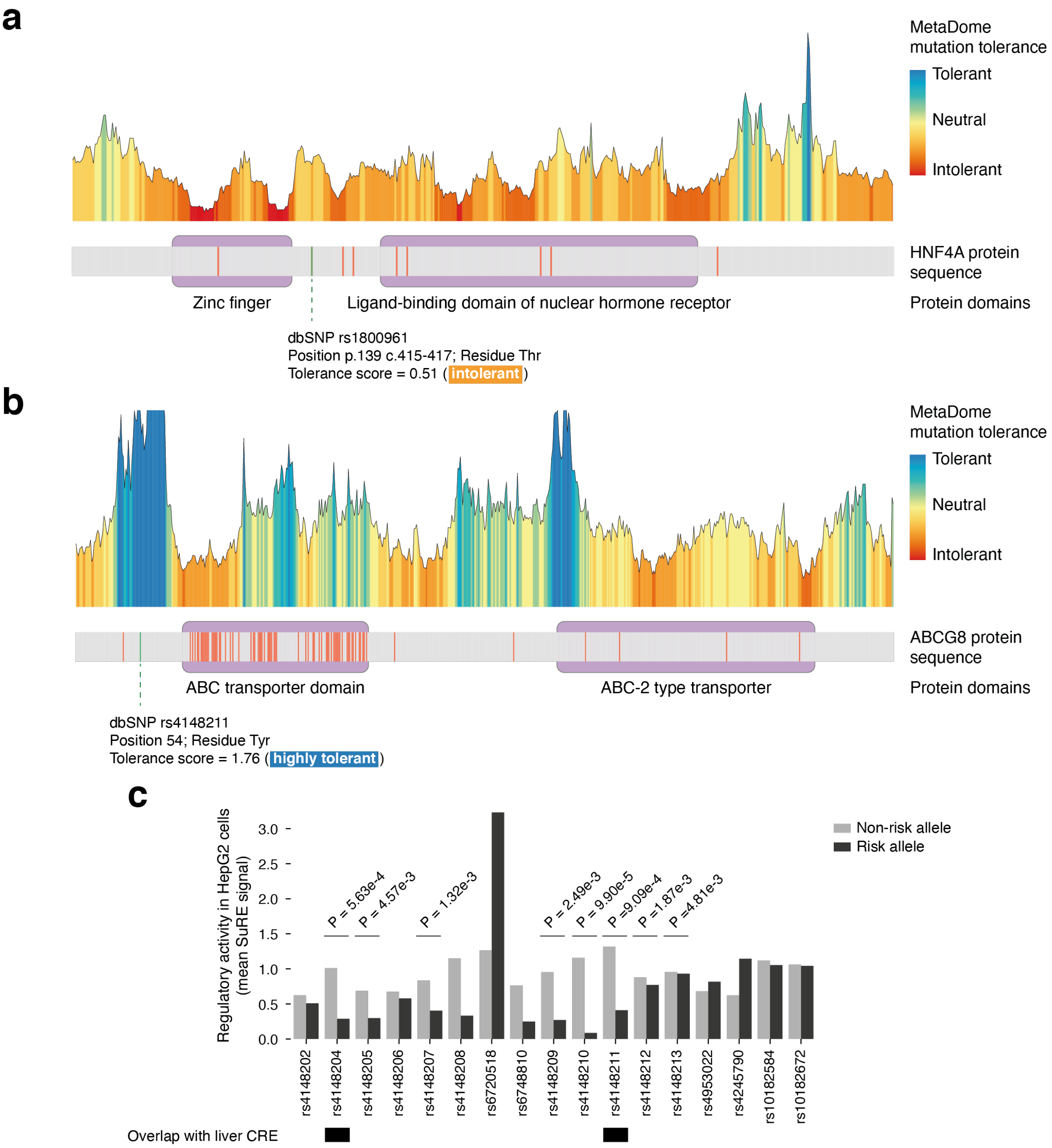

Analysis of the ICP-associated loci *HNF4A* and *ABCG8*. a,b, MetaDome analysis[2] of the missense variants rs1800961 (Thr > Ile) and rs4148211 (Tyr > Cys), which are the lead variants at the *HNF4A* and *ABCG8* loci, respectively. The plots show the position of these two variants relative to meta-domains identified in the HNF4A and ABCG8 proteins. This analysis revealed that rs1800961 is expected to not be tolerated and led us to prioritise this variant as the driver of the association in the *HNF4A* locus. Whereas rs4148211 is expected to be a tolerated sequence change, as it does not affect any meta-domain identified in *ABCG8*. This result suggests that this variant or other variants in high LD with rs4148211 may drive ICP susceptibility via other mechanisms, such as through changing the activity of a cis-regulatory element. **c,** Allele-specific effects of all associated variants in the *ABCG8* locus (EUR $r^2 > 0.8$ with the lead SNP rs4148211) assessed by Survey of Regulatory Elements (SuRE) in HepG2 cells[3]. Mean signal and two-sided Wilcoxon rank-sum test P values comparing the two alleles for each SNP, which were obtained from [3]. P values are only shown for variants that passed the significance threshold of 5% FDR. Source data are provided as a Source Data file.

Supplementary Figure 8

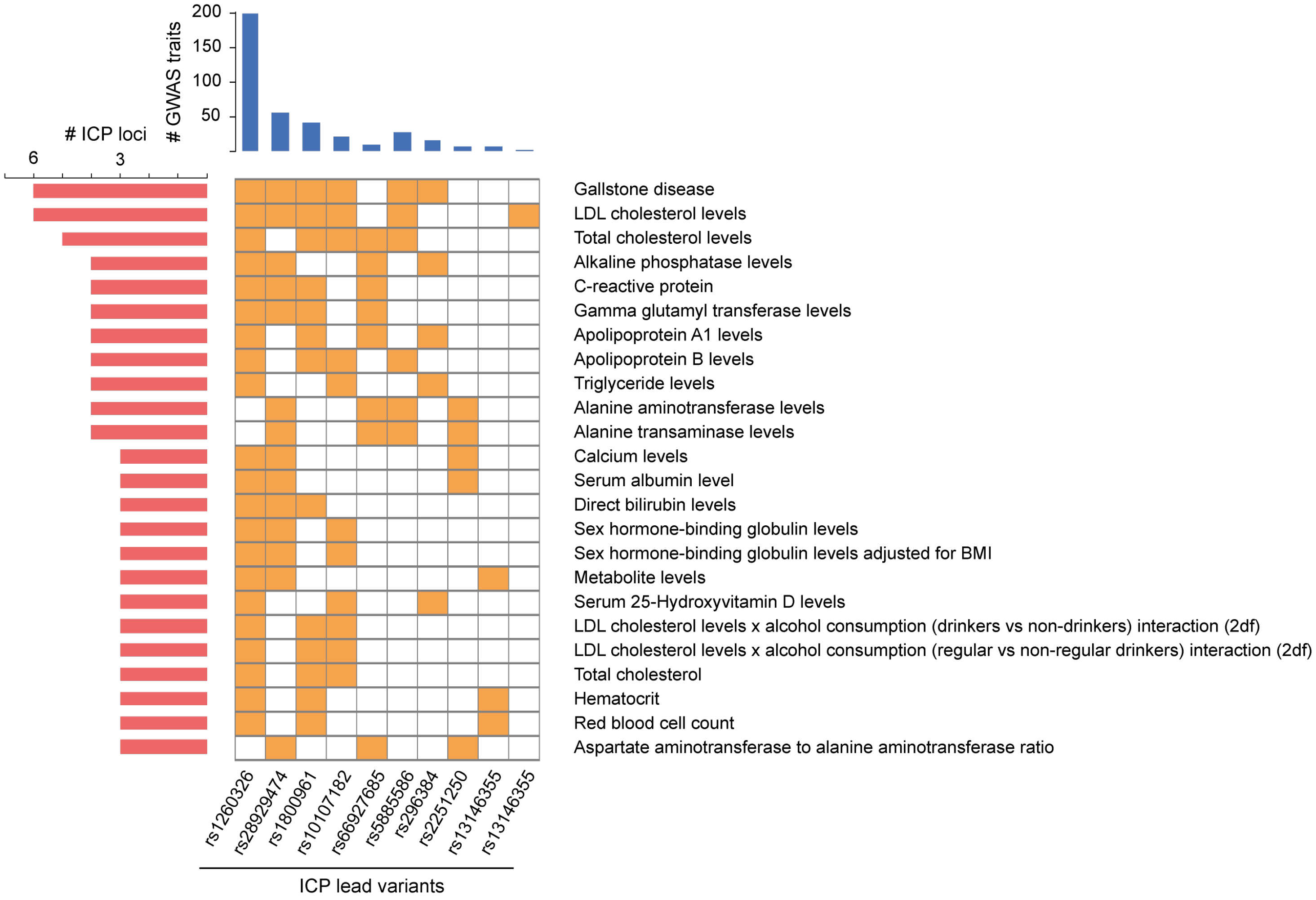

Overlap between genome-wide significant ICP lead variants from the final combined meta-analysis and other traits and diseases reported in the GWAS Catalog. All associations in the GWAS Catalog achieving $p < 5 \times 10^{-6}$ that were in linkage disequilibrium with the 11 identified lead variants at $r^2 > 0.6$ in European populations were included. Only traits/diseases for which we observed an overlap with at least three ICP loci are shwn. The sum of all traits per locus (regardless of being traits with >3 ICP loci) is shown in the blue bar graph on the top. No overlaps were identified for rs34491636.

Supplementary Figure 9

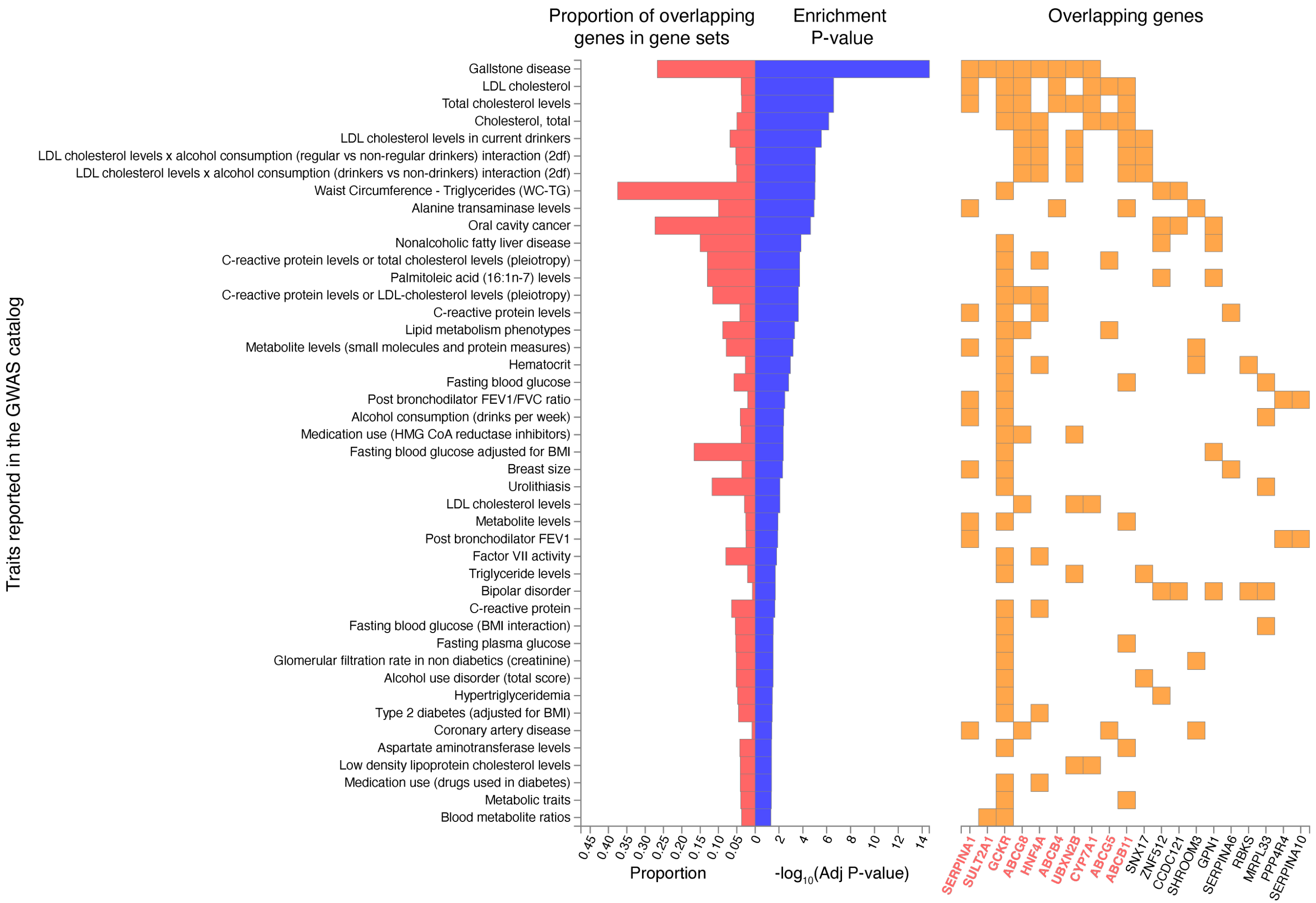

Enrichment analysis of genes in ICP loci for GWAS traits reported in the GWAS Catalog. Genes were selected for inclusion as per Supplementary Figure 5. The genes highlighted in red were prioritised as likely causal of ICP susceptibility after functional genomic analyses. Enrichment analysis was performed using hypergeometric testing with adjustment for multiple comparisons with Bonferroni correction.

Supplementary Note on Methods

NIHR BioResource Rare Disease Collaboration (NIHR-RD)

Study and cohort descriptions

The National Institute for Health Research BioResource Rare Disease Collaboration study (NIHR-RD) involved whole-genome sequencing (WGS) of 13,037 individuals to a clinical standard. A detailed description of the study has been previously published[1]. The study participants included 7,388 individuals assigned to one of 15 rare disease domains, 4,835 individuals recruited as part of the Rare Diseases Pilot of Genomics England, 764 individuals from the UK Biobank with extreme red blood cell indices and 50 control individuals. The 15 rare disease domains include: bleeding / thrombotic / platelet disorders (BPD), cerebral small vessel disease (CSVD), Ehlers-Danlos syndrome (EDS), hypertrophic cardiomyopathy (HCM), intrahepatic cholestasis of pregnancy (ICP), Leber Hereditary Optic Neuropathy (LHON), multiple primary malignant tumours (MPMT), pulmonary arterial hypertension (PAH), primary immune disorders (PID), primary membranoproliferative glomerulonephritis (PMG), inherited retinal disorders (IRD), neurological and developmental disorders (NDD), neuropathic pain disorders (NPD), stem cell and myeloid disorders (SMD) and steroid resistant nephrotic syndrome (SRNS).

The ICP patients were recruited from 14 UK consultant-led antenatal NHS clinics and from three international units in Argentina, Australia, and Sweden. All patients were recruited based on a diagnosis of severe ICP, defined as onset of pruritus prior to 32 weeks gestation and serum bile acids 40μmol/L. Women were excluded from the study if they had other known causes of hepatic dysfunction such as haemolysis, elevated liver enzymes and low platelets (HELLP) syndrome, preeclampsia, acute fatty liver of pregnancy, acute viral hepatitis, confirmed primary biliary cholangitis or any cause of biliary obstruction on ultrasound. No genetic pre-screening for causal variants in known genes was applied before enrolment. Controls were drawn from participants in the non-ICP NIHR-RD cohorts. Individuals in the non-ICP cohorts were not specifically screened to exclude ICP; the prevalence of ICP in these cohorts is not expected to exceed the baseline population prevalence of the disease. None of the other disease domains recruited were expected to have aetiopathological overlap with ICP.

The NIHR-RD study conformed to the guidelines outlined by the 1975 Declaration of Helsinki and permission was obtained from the Ethics Committees of the East of England Cambridge South National Research Ethics Committee (REC 13/EE/0325), the Hammersmith Hospitals NHS Trust, London (REC 97/5197, 08/H0707/21), the Women and Children's Health Network Human Research Ethics Committee South Australia (HREC/15/WCHN/189), the

Swedish Regional Ethics committee (dnr 162-16, 2016-04-20) and the Ramón Sardá Mother's and Children's Hospital ethics committee (MSNSF 07-2016).

Whole-genome sequencing, variant calling and quality control

Full details of the methods employed for whole-genome sequencing (WGS), variant calling and quality control (QC) for the NIHR-RD dataset have been described previously[1,4]. In summary, DNA was extracted from whole blood, underwent QC assessment and was prepared using the Illumina TruSeq DNA PCR-Free sample preparation kit. 100-150 base pair paired-end sequencing was undertaken using an Illumina HiSeq 2500 or HiSeq X. Sequence reads were aligned to the Homo Sapiens NCBI GRCh37 assembly using the Illumina Isaac Aligner[5] (version SAAC00776.15.01.27). The minimum coverage required per sample was at least 95% of the autosomal genome at ≥15X read depth. Single nucleotide variants (SNVs) and indels were called using the Illumina Starling software (version 2.1.4.2 2) and normalized and combined into gVCFs. For each variant, the overall pass rate (OPR) was enumerated as the product of the pass rate (the proportion of alternate genotype passing the original variant filtering) and the call rate (proportion of non-missing genotypes). A genotype quality (GQ) threshold of 20 and depth (DP) threshold of 10 were imposed per genotype per individual; calls failing to meet either of these criteria were set to missing. Sample duplicates (n = 136) and those with poor data quality (n = 14) were excluded. Variants were annotated using the Ensembl Variant Effect Predictor (https://www.ensembl.org/info/docs/tools/vep/index.html) (version 89)[6] and their frequency in gnomAD[7] (https://gnomad.broadinstitute.org/). Variants were retained if they passed the following quality filters: OPR ≥ 0.9, missingness ≤ 0.01, minor allele frequency (MAF) ≥ 0.01, minor allele count (MAC) >10, gnomAD Non-Finnish European (NFE) ≥ 0.01 and ≤ 0.99. Variants were filtered using bcftools[8] (version 1.8) (http://samtools.github.io/bcftools/) and further filtered using PLINK[9,10] (version 1.9) (https://www.cog-genomics.org/plink/) and with custom scripts written in Python (https://www.python.org/) and R (https://www.r-project.org/).

Ancestry and relatedness estimation

A subset of 32,875 high-quality common (MAF ≥ 0.3) autosomal biallelic SNVs in linkage equilibrium ($r^2 < 0.2$) was identified as previously described[1] and genotype data extracted. An initial kinship matrix was computed using KING[11] (https://www.kingrelatedness.com/), PC-AiR[12] and PC-Relate[13] in the R Bioconductor package; GENESIS (https://bioconductor.org/packages/release/bioc/vignettes/GENESIS/inst/doc/pcair.html) were then utilised to correct the kinship matrix for population structure. The resulting kinship matrix was analysed using PRIMUS[14] (https://primus.gs.washington.edu/) to identify the maximal set of unrelated individuals (with relatedness defined by a pairwise kinship coefficient kinship coefficient >

0.09). The ancestry of all samples was estimated with GENESIS by calculating principal components (PC) using unrelated individuals of variable defined ancestry from the 1000 Genomes Project[15] (https://www.internationalgenome.org/) and projecting the NIHR-RD samples onto this vector space. A multivariate model was used to classify each individual's ancestry based on the 1000 Genomes populations. A total of 8,718 unrelated European individuals were identified of which 217 had ICP (**Supplementary Fig. 2a**). A further 66 individuals, including one ICP case, were excluded due to a heterozygosity rate greater or less than 3 standard deviations from the mean (indicating sample contamination or evidence of inbreeding) or missingness > 0.005. The final dataset comprised 216 ICP cases and 8,436 controls.

The 100,000 Genomes Project (100KGP)

Study and cohort descriptions

The Genomics England dataset (https://www.genomicsengland.co.uk/initiatives/100000-genomes-project) consists of whole-genome sequencing (WGS) data, clinical phenotypes encoded using Human Phenotype Ontology (HPO) codes, International Statistical Classification of Diseases and Related Health Problems (ICD) codes, and retrospective and prospectively-ascertained National Health Service (NHS) hospital records for 88,844 individuals recruited with cancer, rare disease, and their unaffected relatives (https://figshare.com/articles/dataset/GenomicEnglandProtocol_pdf/4530893/5). Ethical approval for the 100KGP was granted by the Research Ethics Committee for East of England – Cambridge South (REC Ref 14/EE/1112). ICP was not a targeted recruitment cohort for the 100KGP; however, given the size of the cohort, the population prevalence of ICP and the availability of detailed phenotypic coding, we hypothesised that a number of cases would be present within the dataset. By utilising the ICD-10 code O26.6 (https://icd.who.int/browse10/2019/en#/O26.6) ("Liver disorders in pregnancy, childbirth and the puerperium" including "cholestasis (intrahepatic) in pregnancy" and "obstetric cholestasis") we identified 225 ICP cases. No phenotypic exclusions were applied to the control cohort which consisted of individuals with rare disease, cancer and unaffected relatives without documented evidence of ICP.

Whole-genome sequencing, variant calling and quality control

DNA was extracted from whole blood, underwent QC assessment, and was prepared using the Illumina TruSeq DNA PCR-Free sample preparation kit. 150bp paired-end sequencing was undertaken using an Illumina HiSeq X and processed on the Illumina North Star Version 4 Whole Genome Sequencing Workflow (NSV4, version 2.6.53.23). Sequence reads were aligned to the Homo Sapiens NCBI GRCh38 assembly using the Illumina Isaac Aligner

(version 03.16.02.19). The minimum coverage required per sample was at least 95% of the autosomal genome at ≥15X with mapping quality > 10. SNVs and indels were called using the Illumina Starling software (version 2.4.7). gVCFs were aggregated using Illumina gvcfgenotyper (version 2019.02.26) with variants normalised and multi-allelic variants decomposed using vt (version 0.57721). Variants were retained if they passed the following quality filters: missingness ≤ 0.05, median depth ≥ 10, median GQ ≥ 15, percentage of heterozygous calls not showing significant allele imbalance for reads supporting the reference and alternate alleles (ABratio) ≥ 25%, percentage of complete sites (completeGTRatio) ≥ 50%. Variants were filtered using bcftools[16] (version 1.10.2) and further filtered using PLINK[9,10] (version 2.00).

Ancestry and relatedness estimation

Broad genetic ancestry of the 100KGP samples was estimated using unrelated samples from the 1000 Genomes Project (Phase 3)[15]. A common subset of 62,523 high-quality autosomal biallelic SNVs with MAF > 0.05 in linkage equilibrium ($r^2 < 0.1$) was used to calculate 20 principal components (PCs) using GCTA[17] (version 1.92.4) (https://cnsgenomics.com/software/gcta/#Overview) The 100KGP data were projected onto the 1000 Genomes PC loadings and a random forest model trained to predict ancestries using the first eight PCs. Using this model with a probability threshold > 0.8, 62,349 individuals (183 ICP cases and 62,166 controls) of European ancestry were identified (Supplementary Fig. 2b). A subset of unrelated samples was ascertained using a kinship coefficient threshold of 0.0884 (2nd degree relationships) estimated using KING[11]; one case and 16,336 controls were excluded. A further 245 controls were removed due to a heterozygosity rate greater or less than 3 standard deviations from the mean. A cohort of 182 cases and 45,585 controls remained. The majority of these cases (n = 147, 80.8%) were unaffected relatives of rare disease participants. A smaller number had non-liver related rare disease (n = 23, 12.6%) or cancer (n = 12, 6.6%). Using this final unrelated European cohort, ten principal components were generated using PLINK[10] (version 2.00) for use as covariates in the association analysis.

Supplementary Information References

1. Turro, E. *et al.* Whole-genome sequencing of patients with rare diseases in a national health system. *Nature* **583**, 96-102 (2020).
2. Wiel, L. *et al.* MetaDome: Pathogenicity analysis of genetic variants through aggregation of homologous human protein domains. *Hum Mutat* **40**, 1030-1038 (2019).
3. van Arensbergen, J. *et al.* High-throughput identification of human SNPs affecting regulatory element activity. *Nat Genet* **51**, 1160-1169 (2019).
4. Levine, A.P. *et al.* Large-Scale Whole-Genome Sequencing Reveals the Genetic Architecture of Primary Membranoproliferative GN and C3 Glomerulopathy. *J Am Soc Nephrol* **31**, 365-373 (2020).
5. Raczy, C. *et al.* Isaac: ultra-fast whole-genome secondary analysis on Illumina sequencing platforms. *Bioinformatics* **29**, 2041-3 (2013).
6. McLaren, W. *et al.* The Ensembl Variant Effect Predictor. *Genome Biol* **17**, 122 (2016).
7. Karczewski, K.J. *et al.* The mutational constraint spectrum quantified from variation in 141,456 humans. *Nature* **581**, 434-443 (2020).
8. Danecek, P. *et al.* Twelve years of SAMtools and BCFtools. *Gigascience* **10**(2021).
9. Purcell, S. *et al.* PLINK: a tool set for whole-genome association and population-based linkage analyses. *American journal of human genetics* **81**, 559-75 (2007).
10. Chang, C.C. *et al.* Second-generation PLINK: rising to the challenge of larger and richer datasets. *Gigascience* **4**, 7 (2015).
11. Manichaikul, A. *et al.* Robust relationship inference in genome-wide association studies. *Bioinformatics* **26**, 2867-73 (2010).
12. Conomos, M.P., Miller, M.B. & Thornton, T.A. Robust inference of population structure for ancestry prediction and correction of stratification in the presence of relatedness. *Genet Epidemiol* **39**, 276-93 (2015).
13. Conomos, M.P., Reiner, A.P., Weir, B.S. & Thornton, T.A. Model-free Estimation of Recent Genetic Relatedness. *Am J Hum Genet* **98**, 127-48 (2016).
14. Staples, J., Nickerson, D.A. & Below, J.E. Utilizing graph theory to select the largest set of unrelated individuals for genetic analysis. *Genet Epidemiol* **37**, 136-41 (2013).
15. Genomes Project, C. *et al.* A global reference for human genetic variation. *Nature* **526**, 68-74 (2015).
16. Li, H. *et al.* The Sequence Alignment/Map format and SAMtools. *Bioinformatics* **25**, 2078-9 (2009).
17. Yang, J., Lee, S.H., Goddard, M.E. & Visscher, P.M. GCTA: a tool for genome-wide complex trait analysis. *Am J Hum Genet* **88**, 76-82 (2011).
