# Supplementary material for: GWAS meta-analysis of intrahepatic cholestasis of pregnancy implicates multiple hepatic genes and regulatory elements
Source: Nat Commun. 2022 Aug 17;13:4840. doi: 10.1038/s41467-022-29931-z (PMC9385867; doi:10.1038/s41467-022-29931-z)
Supplement: Supplementary file 3 — Description of Additional Supplementary Files [file 41467_2022_29931_MOESM3_ESM.pdf]

Description of Additional Supplementary Files

File Name: Supplementary Data 1
Description: GWAS and GWAS meta-analysis summary statistics for the lead variants.

File Name: Supplementary Data 2
Description: Variant prioritization using PAINTOR.

File Name: Supplementary Data 3
Description: Summary of coding variants identified in the ICP GWAS meta-analysis

File Name: Supplementary Data 4
Description: Full data of the overlap between genome-wide significant ICP lead variants from the final combined meta-analysis and other traits and diseases reported in the GWAS Catalog

File Name: Supplementary Data 5
Description: Coordinates of human adult liver accessible chromatin regions (GRCh38/hg38)
