# Supplementary material for: GWAS meta-analysis of intrahepatic cholestasis of pregnancy implicates multiple hepatic genes and regulatory elements
Source: Nat Commun. 2022 Aug 17;13:4840. doi: 10.1038/s41467-022-29931-z (PMC9385867; doi:10.1038/s41467-022-29931-z)
Supplement: Supplementary file 5 — Reporting Summary [file 41467_2022_29931_MOESM5_ESM.pdf]

nature research

Corresponding author(s): Catherine Williamson

Last updated by author(s): March 9 2022

Reporting Summary

Nature Research wishes to improve the reproducibility of the work that we publish. This form provides structure for consistency and transparency in reporting. For further information on Nature Research policies, see our Editorial Policies and the Editorial Policy Checklist.

Statistics

For all statistical analyses, confirm that the following items are present in the figure legend, table legend, main text, or Methods section.

n/a	Confirmed	
☐	☒	The exact sample size (n) for each experimental group/condition, given as a discrete number and unit of measurement
☐	☒	A statement on whether measurements were taken from distinct samples or whether the same sample was measured repeatedly
☐	☒	The statistical test(s) used AND whether they are one- or two-sided<br>*Only common tests should be described solely by name; describe more complex techniques in the Methods section.*
☐	☒	A description of all covariates tested
☐	☒	A description of any assumptions or corrections, such as tests of normality and adjustment for multiple comparisons
☐	☒	A full description of the statistical parameters including central tendency (e.g. means) or other basic estimates (e.g. regression coefficient) AND variation (e.g. standard deviation) or associated estimates of uncertainty (e.g. confidence intervals)
☐	☒	For null hypothesis testing, the test statistic (e.g. F, t, r) with confidence intervals, effect sizes, degrees of freedom and P value noted<br>*Give P values as exact values whenever suitable.*
☒	☐	For Bayesian analysis, information on the choice of priors and Markov chain Monte Carlo settings
☒	☐	For hierarchical and complex designs, identification of the appropriate level for tests and full reporting of outcomes
☐	☒	Estimates of effect sizes (e.g. Cohen's d, Pearson's r), indicating how they were calculated

Our web collection on statistics for biologists contains articles on many of the points above.

Software and code

Policy information about availability of computer code

Data collection	None
Data analysis	Illumina Isaac Aligner (version SAAC00776.15.01.27 and 03.16.02.19), Illumina Starling software (version 2.1.4.2 and 2.4.7), Ensembl Variant Effect Predictor (versions 89 and 104), bcftools (version 1.8 and 1.10.2), PLINK (version 1.07, 1.9 and 2.0), Illumina North Star Version 4 Whole Genome Sequencing Workflow (NSV4, v2.6.53.23), Illumina Isaac Aligner (version 03.16.02.19), Illumina Starling software (version 2.4.7), Illumina gvcfgenotyper (version 2019.02.26), vt (version 0.57721), CrossMap (version 0.5.2), METAL (version 2011-03-25), LocusZoom (version 0.13.3), GCTA (version 1.92.4 and 1.93.2), LDlinkR (version 1.1.2), KING, PRIMUS, GENESIS (PC-Air, PC-Relate), FUMA (version 1.3.6b), LDlink (LDtrait Tool), PAINTOR (version 3.1), SAIGE (versions 0.35.8.8, 0.42.1 and 0.44.2), qqman (version 0.1.8), genpwr (version 1.0.4), Motifbreak R (version 2.2.0), Mangrove (version 1.21), Python packages: pybedtools (version 0.8.0) and seaborn (version 0.11.2). Version numbers have been provided where available/applicable.

For manuscripts utilizing custom algorithms or software that are central to the research but not yet described in published literature, software must be made available to editors and reviewers. We strongly encourage code deposition in a community repository (e.g. GitHub). See the Nature Research guidelines for submitting code & software for further information.

Data

Policy information about availability of data

All manuscripts must include a data availability statement. This statement should provide the following information, where applicable:

- Accession codes, unique identifiers, or web links for publicly available datasets
- A list of figures that have associated raw data
- A description of any restrictions on data availability

The genome-wide summary statistics for the final combined meta-analysis carried out in this study have been deposited in the GWAS Catalog under accession code

GCP000309 [hyperlink will be provided in accepted version]. The lead variants' GWAS and GWAS meta-analysis summary statistics generated in this study are provided in the Supplementary Table 1. Genotype and phenotype data from the NIHR-RD participants are available from several sources. 4,835 of the NIHR-RD participants were part of the 100,000 Genomes Project – Rare Diseases Pilot. These data can be accessed by application to Genomics England Limited following the procedure outlined at https://www.genomicsengland.co.uk/about-gecip/joining-researchcommunity/. The genotype and phenotype data from the remaining 7,348 NIHR-RD participants can be accessed by application to the NIHR BioResource Data Access Committee at dac@bioresource.nihr.ac.uk. Subject to ethical consent, the genotype data of a subset of 6,939 NIHR-RD participants are also available from the European Genome-phenome Archive (EGA) at the EMBL European Bioinformatics Institute [https://ega-archive.org/dacs/EGAC00001000259]. This includes data from 305 ICP cases [https://ega-archive.org/datasets/EGAD00001004515]. Genomic and phenotype data from the 100KGP participants can be accessed by application to Genomics England Limited following the procedure outlined at https://www.genomicsengland.co.uk/about-gecip/joining-researchcommunity/. Genotype data for the 764 UK Biobank samples are available through the UK Biobank [https://www.ukbiobank.ac.uk/]. The FinnGenn GWAS summary statistics are publicly accessible following registration [https://www.finngen.fi/en/access_results]. The adult human liver replicated accessible regions generated in this study are provided in the Supplementary Table 5. The variant pathogenicity data used in this study are available in the ClinVar database [https://www.ncbi.nlm.nih.gov/clinvar/] and through MetaDome v1.0.1 [https://stuart.radboudumc.nl/metadome]. The CADD scores (v1.6) used in this study are available in the CADD website [https://cadd.gs.washington.edu/download]. The liver epigenomic and transcriptomic data used in this study are available in the ENCODE portal [https://www.encodeproject.org/]. The SuRE data used in this study are available in the OFS data repository [https://osf.io/pjxm4/]. The transcription factor motif logos used in this study are available in the JASPAR database [http://jaspar.genereg.net/]. The eQTL data used in this study are available in the Genotype-Tissue Expression (GTEx) Portal under dbGaP accession phs000424.v8.p2 [https://gtexportal.org/home/]. The tissue specificity SPECS scores for liver tissue used in this study are available in the SPECS web browser [https://specs.cmgg.be]. The adult liver TAD coordinates used in this study are available in the 3D Genome Browser [http://3dgenome.fsm.northwestern.edu/index.html].

In addition to the GWAS summary statistics, we provide Source Data for Figure 4b, Figure 7c and Supplementary Figure 7c.

Field-specific reporting

Please select the one below that is the best fit for your research. If you are not sure, read the appropriate sections before making your selection.

- [x] Life sciences
- [ ] Behavioural & social sciences
- [ ] Ecological, evolutionary & environmental sciences

For a reference copy of the document with all sections, see nature.com/documents/nr-reporting-summary-flat.pdf

Life sciences study design

All studies must disclose on these points even when the disclosure is negative.

Sample size	All available samples passing QC were included to maximise power.
Data exclusions	Samples failing quality control were excluded, as detailed in the supplementary information.
Replication	Evidence of replication was observed with meta-analysis of the three independent datasets.
Randomization	This is a genetic association study and randomization is not applicable to this methodology.
Blinding	This is a genetic association study and blinding is not applicable to this methodology.

Reporting for specific materials, systems and methods

We require information from authors about some types of materials, experimental systems and methods used in many studies. Here, indicate whether each material, system or method listed is relevant to your study. If you are not sure if a list item applies to your research, read the appropriate section before selecting a response.

Materials & experimental systems

n/a	Involved in the study	
x		Antibodies
x		Eukaryotic cell lines
x		Palaeontology and archaeology
x		Animals and other organisms
	x	Human research participants
x		Clinical data
x		Dual use research of concern

Methods

n/a	Involved in the study	
x		ChIP-seq
x		Flow cytometry
x		MRI-based neuroimaging

Human research participants

Policy information about studies involving human research participants

Population characteristics	Both males and females of European ancestry were included. Cases were individuals with a diagnosis of intrahepatic cholestasis of pregnancy. Controls were either healthy individuals or individuals recruited to studies with other diseases. Further details are provided in the Supplementary Information. Additional phenotype information (e.g., regarding age) is not available or relevant to ICP.
Recruitment	Data were taken from three previously described studies: The National Institute for Health Research BioResource Rare Disease Collaboration Study, The 100,000 Genomes Project and The FinnGen Study.
Ethics oversight	The NIHR-RD study conformed to the guidelines outlined by the 1975 Declaration of Helsinki and permission was obtained from the Ethics Committees of the East of England Cambridge South National Research Ethics Committee (REC 13/EE/0325), the Hammersmith Hospitals NHS Trust, London (REC 97/5197, 08/H0707/21), the Women and Children's Health Network Human Research Ethics Committee South Australia (HREC/15/WCHN/189), the Swedish Regional Ethics committee (dnr 162-16, 2016-04-20) and the Ramón Sardá Mother's and Children's Hospital ethics committee (MSNSF 07-2016).<br><br>Ethical approval for the 100KGP was granted by the Research Ethics Committee for East of England – Cambridge South (REC Ref 14/EE/1112).

Note that full information on the approval of the study protocol must also be provided in the manuscript.
